# Ex vivo exposure of bone marrow from chronic kidney disease donor rats to pravastatin limits renal damage in recipient rats with chronic kidney disease

**DOI:** 10.1186/s13287-015-0064-7

**Published:** 2015-04-15

**Authors:** Arianne van Koppen, Diana A Papazova, Nynke R Oosterhuis, Hendrik Gremmels, Rachel H Giles, Joost O Fledderus, Jaap A Joles, Marianne C Verhaar

**Affiliations:** Department of Nephrology & Hypertension, University Medical Center Utrecht, F03.223, Heidelberglaan 100, Utrecht, 3584 CX The Netherlands; Department of Metabolic Health Research, The Netherlands Organization for Applied Scientific Research (TNO), Zernikedreef 9, 2333 CK, Leiden, The Netherlands

## Abstract

**Introduction:**

Healthy bone marrow cell (BMC) infusion improves renal function and limits renal injury in a model of chronic kidney disease (CKD) in rats. However, BMCs derived from rats with CKD fail to retain beneficial effects, demonstrating limited therapeutic efficacy. Statins have been reported to improve cellular repair mechanisms.

**Methods:**

We studied whether exposing CKD rat BMCs *ex vivo* to pravastatin improved their *in vivo* therapeutic efficacy in CKD and compared this to systemic *in vivo* treatment. Six weeks after CKD induction, healthy BMCs, healthy pravastatin-pretreated BMCs, CKD BMCs or CKD pravastatin-pretreated BMCs were injected into the renal artery of CKD rats.

**Results:**

At 6 weeks after BMC injection renal injury was reduced in pravastatin-pretreated CKD BMC recipients vs. CKD BMC recipients. Effective renal plasma flow was lower and filtration fraction was higher in CKD BMC recipients compared to all groups whereas there was no difference between pravastatin-pretreated CKD BMC and healthy BMC recipients. Mean arterial pressure was higher in CKD BMC recipients compared to all other groups. In contrast, 6 weeks of systemic *in vivo* pravastatin treatment had no effect. *In vitro* results showed improved migration, decreased apoptosis and lower excretion of pro-inflammatory Chemokine (C-X-C Motif) Ligand 5 in pravastatin-pretreated CKD BMCs.

**Conclusions:**

Short *ex vivo* exposure of CKD BMC to pravastatin improves CKD BMC function and their subsequent therapeutic efficacy in a CKD setting, whereas systemic statin treatment did not provide renal protection.

## Introduction

The rapidly increasing number of patients with chronic kidney disease (CKD) worldwide urgently calls for new interventions. Bone marrow (BM)-derived stem and progenitor cell-based therapies have been proposed as a promising approach for the treatment of acute kidney disease and CKD. We recently demonstrated that administration of healthy donor bone marrow cells (BMCs) in an established rat model of CKD reduced progression of CKD [1]. However, for clinical application of BMC therapy in CKD, the use of autologous BMCs would be preferred; the challenge is that BMCs derived from CKD donors are unable to recapitulate therapeutic efficacy [1]. Therefore, to optimize autologous BMC therapy for treatment of CKD, we aim to counteract the functional impairment of CKD BMCs.

Several investigators have reported strategies to improve function of autologous BM-derived progenitor cell populations. Systemic treatment with lipid-lowering 3-hydroxy-3-methyl-glutaryl Coenzyme A reductase (HMG-CR) inhibitors (statins) has been shown to augment endothelial progenitor cell (EPC) and mesenchymal stem cell (MSC) number and function in several disease models, including cardiovascular diseases [2-4] and hypertension [5,6]. *In vitro* statin incubation has been reported to improve cell differentiation, proliferation, migration, angiogenesis and adhesion and to decrease senescence, apoptosis and inflammation [7,8], possibly by pleiotropic effects such as increased nitric oxide (NO) bioavailability, anti-inflammatory and antioxidant effects [2,3,8,9] and prevention of cellular senescence via regulation of cell cycle proteins [10].

However, few studies have investigated the effect of *in vitro* or *ex vivo* statin treatment on cells obtained from diseased cell source [11-16], and to the best of our knowledge this is the first study to report the effects of statin treatment on cells in the context of CKD. We hypothesized that exposing CKD BMCs to pravastatin *ex vivo* would improve their subsequent *in vivo* therapeutic efficacy, ameliorating the progression of renal failure in a rat model of established CKD. To this end, we studied long-term effects of intra-arterial delivery of vehicle-pretreated or pravastatin-pretreated healthy and CKD BMCs on renal hemodynamics and injury. Our data conclude that while systemic *in vivo* treatment with pravastatin does not influence the course of CKD, a short *ex vivo* pulse of pravastatin significantly ameliorates progression of CKD in an established rat model.

## Methods

### *In vitro* experiments

BMCs were harvested at 6 weeks after CKD induction from CKD Lewis rats (for characteristics, see Table 1) and were incubated with or without 1 mmol/l pravastatin in Dulbecco’s modified Eagle medium (DMEM) for 2 hours at 37°C. Directly after incubation, cells were centrifuged and the conditioned medium was stored for further analysis. Cells were washed, resuspended in 1 ml DMEM and used for assessment of *in vitro* migration and apoptosis. Then 1 × 10^6^ cells were stored in Trizol (Invitrogen, Grand Island, NY, USA) for RNA extraction.

**Table 1:** **Donor characteristics and stratification of donor and recipient rats**

***In vitro*** **pravastatin treatment**	**CKD (** ***n*** **= 10)**			
SNX (%)	66 ± 2			
Week 5 SBP (mmHg)	169 ± 28			
Week 5 urea (mmol/l)	11 ± 2			
Week 5 proteinuria (mg/24 hours)	18 ± 28			
***Ex vivo*** **pravastatin treatment experiment**				
*Donor rats*	**Healthy + DMEM BMC (** ***n*** **= 2)**	**Healthy + pravastatin BMC (** ***n*** **= 2)**	**CKD + DMEM BMC (** ***n*** **= 4)**	**CKD + pravastatin BMC (** ***n*** **= 5)**
SNX (%)	–	–	64.0 ± 6.0	69.0 ± 5.5
Week 5 SBP (mmHg)	–	–	148 ± 34	152 ± 8
Week 5 urea (mmol/l)	4.6 ± 0.01	4.1 ± 0.9	15.0 ± 4.4	15.9 ± 6.9
Week 5 proteinuria (mg/24 hours)	1.4 ± 0.4	1.8 ± 0.7	24 ± 8	49 ± 46
*Recipient rats*	**Healthy + DMEM BMC (** ***n*** **= 5)**	**Healthy + pravastatin BMC (** ***n*** **= 5)**	**CKD + DMEM BMC (** ***n*** **= 10)**	**CKD + pravastatin BMC (** ***n*** **= 9)**
SNX (%)	66.3 ± 1.6	66.7 ± 6.4	66.5 ± 3.5	67.6 ± 7.1
Week 5 SBP (mmHg)	156 ± 15	155 ± 16	159 ± 25	155 ± 19
Week 5 urea (mmol/l)	10.1 ± 0.9	10.3 ± 1.5	12.1 ± 3.2	10.6 ± 2.3
Week 5 proteinuria (mg/24 hours)	21.6 ± 5.6	21.9 ± 6.8	23.7 ± 17.3	22.7 ± 8.4
**Systemic** ***in vivo*** **pravastatin treatment**				
*Recipient rats*	**CKD + pravastatin (** ***n*** **= 5)**	**CKD (** ***n*** **= 6)**		
SNX (%)	69 ± 2	66 ± 3		
Week 5 SBP (mmHg)	158 ± 19	159 ± 10		
Week 5 urea (mmol/l)	12.7 ± 1.1	12.8 ± 1.4		
Week 5 proteinuria (mg/24 hours)	14.6 ± 2.7	16.5 ± 6.6		

At 1 week before BMC or *in vivo* pravastatin administration (week 5 after SNX), rats were stratified based on plasma urea and SBP. Data presented as mean ± standard deviation. BMC, bone marrow cell; CKD, chronic kidney disease; DMEM, Dulbecco’s modified Eagle medium; SBP, systolic blood pressure; SNX, subtotal nephrectomy.

### Bone marrow cell characteristics

Viability and proportions of myeloid and lymphoid cell precursors were studied by flow cytometry.

### Migration assay

Migration of pretreated CKD BMCs was determined using a modified Boyden chamber assay. Then 300,000 living cells were loaded above a 5 μm polycarbonate membrane (transwell permeable support system; Corning, Tewksbury, MA, NY, USA) and the wells below contained 200 ng/ml stromal derived factor 1 alpha (SDF1α), a strong BMC attractant [17]; vehicle (no SDF1α) was used as negative control. After 180 minutes, transwells were removed horizontally and 1 ml of 2 mmol/l phosphate-buffered saline-ethylenediamine tetraacetic acid was added to the bottom well and incubated for 15 minutes on ice. Cell suspensions were collected and counted by flow cytometry. The percentage of migrated DMEM-treated BMCs towards 200 ng/ml SDF1α was set at 100% and compared with the migration of pravastatin-treated cells.

### Apoptosis

Cell suspension (50 μl) was used to create a cell smear, air dried, fixed with formalin and stored at −20°C. Terminal deoxynucleotidyl transferase dUTP nick end labeling (TUNEL) staining (Apoptag Plus in situ Peroxidase kit; Millipore, Temecula, CA, USA) was performed according to the manufacturer’s guidelines. The number of apoptotic cells was determined as the number of TUNEL-positive cells in the images of 20 randomly selected fields (×200 magnification).

### Quantitative real-time PCR

We performed quantitative real-time PCR to determine the effects of pretreatment of CKD BMCs with pravastatin on the mRNA expression of endothelial NO synthase, protein kinase B, monocyte chemotactic protein 1, tumor necrosis factor alpha and vascular endothelial growth factor (ABiPRiSM 790Sequence Detection System; Applied Biosystems, Foster City, CA, USA). The following TaqMan^®^ Gene Expression Assays (Applied Biosystems) were used: endothelial NO synthase, Rn02132634_s1; protein kinase B, Rn00583646_m1; monocyte chemotactic protein 1, Rn00580555_m1; tumor necrosis factor alpha, Rn99999017_m1; vascular endothelial growth factor, Rn00582935_m1; β-actin, Rn00667869_m1; and calnexin, Rn00596877_m1. Reactions were carried out in duplicate. Cycle time values for genes of interest were normalized for mean cycle time values of Calnexin and β-actin, which we previously determined to be the two most stable housekeeping genes across all groups using the geNorm program [18], and expressed relative to a calibrator (the DMEM group), using the ΔΔCt method. Hence, steady-state mRNA levels were expressed as the *n*-fold difference relative to the calibrator.

### Cytokine array

A rat cytokine array (R&D Systems, Minneapolis, MN, USA) of 27 cytokines was performed according to the manufacturer’s instructions on conditioned medium of BMC samples obtained from six CKD rats pretreated in DMEM with or without 1 mmol/l pravastatin as reported previously [1,19]. Equal amounts of protein were loaded on the blots. Each spot on the blot is represented in duplicate and averages of the two pixel densities were used to calculate the average pixel density with Image J software (Rasband W.S. ImageJ, National Institute of Health, Bethesda, Maryland, USA). Background staining and spot size were analyzed as recommended by the manufacturer. In brief, images were converted to 8-bit inverted tagged image file format files and spots were circled. Per blot, equal spot sizes were analyzed.

### Enzyme-linked immunosorbent assay

An enzyme-linked immunosorbent assay for chemokine (C-X-C motif) ligand (CXCL) 5 (Sigma, St. Louis, MO, USA) was performed according to the manufacturer’s instructions on conditioned medium of BMC samples obtained from six CKD rats pretreated in DMEM with or without 1 mmol/l pravastatin to validate cytokine array results.

### *In vivo* experiments

#### Chronic kidney disease induction

The study protocol was approved by the Utrecht University Committee of Animal Experiments. CKD was induced in 8-week-old inbred male Lewis rats (recipients) and enhanced green fluorescent protein (eGFP)^+^ Lewis rats (donors) by two-stage subtotal nephrectomy as described previously (*t* = 0) [1,19]. At week 5, CKD was confirmed (plasma urea >9 mmol/l).

### Experimental design

#### Effects of *ex vivo* pravastatin-pretreated BMCs in established CKD

At week 5 after CKD induction recipient rats (*n* = 29) were stratified based on plasma urea and systolic blood pressure (Table 1). At 6 weeks after subtotal nephrectomy, BMCs were harvested from the femur and tibia of healthy or CKD eGFP^+^ donor rats (for donor characteristics, see Table 1) and suspended in DMEM. The cell suspension was filtered (100 μm sieve) and counted (Abbott Cell-Dyn 1800; Abbott Laboratories, Abbott Park, Illinois, USA). BMCs were incubated with or without 1 mmol/l pravastatin in DMEM for 2 hours at 5% carbon dioxide in a humidified incubator at 37°C. Cells were washed to remove pravastatin and resuspended in 500 μl DMEM. Then 50 × 10^6^*ex vivo* pretreated BMC cells were injected directly into the remnant kidney via the renal artery as follows: healthy + DMEM BMC recipients (*n* = 5), CKD rats injected with healthy eGFP^+^BMC exposed to DMEM; healthy + pravastatin BMC recipients (*n* = 5), CKD rats injected with healthy eGFP^+^BMC exposed to pravastatin; CKD + DMEM BMC recipients (*n* = 10), CKD rats injected with CKD eGFP^+^BMC exposed to DMEM; and CKD + pravastatin BMC recipients (*n* = 9), CKD rats injected with CKD eGFP^+^BMC exposed to pravastatin. Longitudinal measurements were performed at weeks 7, 9 and 11 after subtotal nephrectomy, and at week 12 (6 weeks after BMC injection) terminal kidney function was measured (see Terminal kidney function). Directly thereafter, rats were sacrificed and tissues were collected and either frozen or fixed in 4% paraformaldehyde for renal morphology measurements.

#### Effects of systemic *in vivo* pravastatin treatment in established CKD

At week 6 after CKD induction, CKD rats were divided into two groups: CKD (*n* = 6), no supplement in drinking water; and CKD + pravastatin (*n* = 5), 50 mg/kg/day pravastatin added to drinking water. Longitudinal measurements were performed at weeks 7, 9 and 11, and at week 12 terminal kidney function was measured (see Terminal kidney function). Directly thereafter, rats were sacrificed and tissues were collected and either frozen or fixed in 4% paraformaldehyde for renal morphology measurements.

### Longitudinal chronic kidney disease evaluation

Rats were weighed weekly, and at regular intervals 24-hour urine and blood samples were collected and the systolic blood pressure was measured by tail cuff sphygmomanometry at weeks 5, 9 and 11. To collect 24-hour urine, rats were placed in metabolism cages without food for 24 hours, but with free access to water with 2% glucose. For the systemic *in vivo* pravastatin treatment studies, pravastatin was also supplemented to the drinking water during urine collection. Urine was collected in antibiotic/antimycotic solution (A5955; Sigma) and stored at −80°C. Blood samples were collected from the tail vein. Urinary protein levels were measured with Coomassie blue. Sodium and potassium levels were determined by flame photometry. NO metabolites were measured (Cayman Chemical, Ann Arbor, MI, USA). Plasma urea and plasma and urinary creatinine levels were determined by DiaSys Urea CT FS (DiaSys Diagnostic Systems, Holzheim, Germany). Creatinine clearance was calculated by dividing urine creatinine excretion (μmol/minute/100 g body weight) by plasma creatinine (μmol/μl). Cholesterol and triglycerides were determined by DiaSys Cholesterol FS and DiaSys Triglycerides FS (DiaSys Diagnostic Systems).

### Terminal kidney function

Kidney function was assessed by inulin clearance to determine the glomerular filtration rate (GFR) and by para-amino hippuric acid clearance to determine the effective renal plasma flow (ERPF) as described previously [20]. Briefly, rats were anesthetized with isoflurane and placed on a servo-controlled surgical table to maintain body temperature at 37°C. A polyethylene-90 catheter was placed in the left jugular vein for infusion of solutions. The left femoral artery was cannulated with polyethylene-50 tubing for measurement of mean arterial pressure and blood sampling. A polyethylene-90 catheter was placed in the bladder for urine collection. During surgery, animals received an intravenous infusion of a 150 mmol/l NaCl solution containing 6% bovine serum albumin. Following surgery, the infusion was switched to a 150 mmol/l NaCl solution with 1% bovine serum albumin at the same infusion rate. This infusion was maintained throughout the experiment. The solution also contained inulin and para-amino hippuric acid for clearance measurements. A 60-minute equilibration period was observed before the start of the 60-minute clearance measurements. During this clearance, measurement urine was sampled for 15-minute periods and both before and after the clearance measurement blood was sampled. Clearance and fractional excretions were calculated by standard formulas. Renal blood flow was calculated from ERPF and hematocrit.

### Renal and cardiac morphology

Focal glomerulosclerosis and tubular interstitial damage were scored on 3 μm periodic acid Schiff-stained paraffin-embedded slides [1,19]. Glomerular influx of donor BMCs (eGFP^+^) and the presence of T cells (CD3^+^), monocytes/macrophages (ED-1^+^), proliferating cells (Ki67^+^), apoptotic cells (TUNEL^+^), cells undergoing DNA damage repair (γH2AX^+^) and endothelial cells (JG12^+^) was counted in 50 glomeruli [21]. The tubular number of T cells (CD3^+^), apoptotic cells (TUNEL^+^) and cells undergoing DNA damage repair (γH2AX^+^) and influx of donor BMCs (GFP^+^) were determined in 20 tubular fields as described previously [1,19]. Cardiac collagen I and collagen III contents were stained with Sirius red, visualized with circular polarized light and digitally analyzed using ImageJ software [22]. The percentage of collagen area was calculated by dividing the Sirius red stained area by the total image area.

### Blood and bone marrow characteristics

Blood and BM were incubated with the cell-permeable DNA binding dye Vybrant^®^ DyeCycle™ Violet (Invitrogen/Life, Bleiswijk, the Netherlands) for 30 minutes at 37°C to allow separation of nucleated cells from debris. Red blood cells were then lysed by incubation with a 0.8% ammonium chloride lysis solution and samples were washed with phosphate-buffered saline prior to flow cytometry. During flow cytometry, debris and dead cells were excluded by lack of DyeCycle binding and 7-aminoactinomycin D (Invitrogen) staining, respectively. The remaining cells were divided into major leukocyte subpopulations (that is, lymphocytes, monocytes, granulocytes) on the basis of low-angle forward scatter and orthogonal (‘side’) scatter properties. Gates for the fluorescent stains and eGFP were set on fluorescence-minus-one controls, and gates for leukocyte subpopulations were set for each population center on the contour plot. A volumetric estimate of leukocyte subpopulation proportions was furthermore obtained using an automated hematology analyzer (Abbott Cell-Dyn 1800).

### Quantitative real-time PCR

To ascertain that the dose of pravastatin was sufficient, the production of HMGCR was evaluated. cDNA was isolated from frozen liver tissue and expression of HMGCR (Rn00565598_m1) was determined using quantitative real-time RT-PCR as described for the *in vitro* experiments.

### Statistical analyses

Data are presented as mean ± standard deviation and analyzed by analysis of variance (one-way analysis of variance with a Student–Newman–Keuls post test, two-way analysis of variance with a Bonferroni post test, and Student’s *t* test) with Graphpad Prism software (GraphPad, La Jolla, CA, USA). *P* <0.05 was considered significant.

## Results

### *In vitro* experiments

#### *In vitro* pravastatin pre-treatment of BMCs improves BMC function

**Figure 1:**
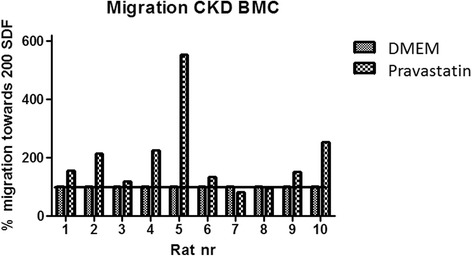
Effect of *in vitro* pravastatin treatment on migration capacity of chronic kidney disease bone marrow cells. Dulbecco’s modified Eagle medium (DMEM; *n* = 10); pravastatin (*n* = 10). BMC, bone marrow cell; CKD, chronic kidney disease; SDF, stromal derived factor.

**Figure 2:**
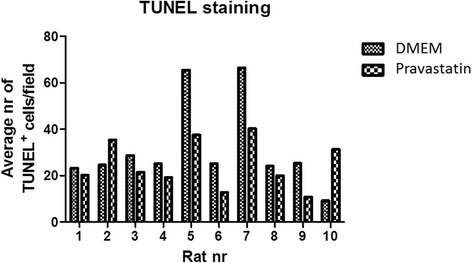
Effect of *in vitro* pravastatin treatment on apoptosis in chronic kidney disease bone marrow cells. Dulbecco’s modified Eagle medium (DMEM; *n* = 10); pravastatin (*n* = 10). TUNEL, Terminal deoxynucleotidyl transferase dUTP nick end labeling.

**Figure 3:**
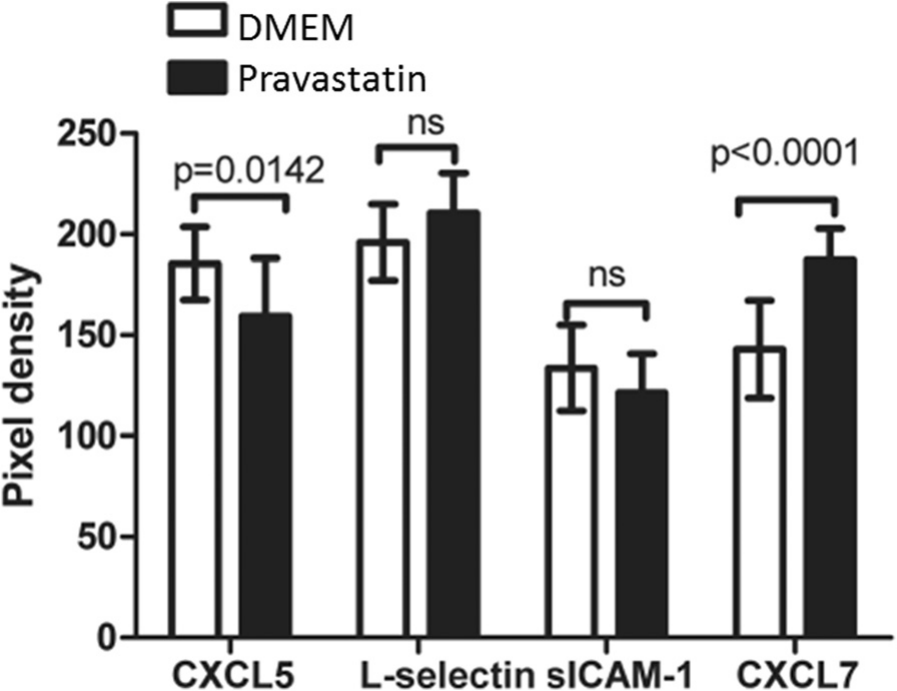
Cytokine expression in chronic kidney disease bone marrow cells after exposure to vehicle (DMEM) or pravastatin *in vitro*. Dulbecco’s modified Eagle medium (DMEM; *n* = 6); pravastatin (*n* = 6). CXCL, chemokine (C-X-C motif) ligand; sICAM, soluble intracellular adhesion molecule.

Pravastastin treatment of CKD BMC did not induce differences in myeloid and lymphoid precursor cell composition (Table 2). CKD + pravastatin BMCs showed increased migration towards 200 ng/ml SDF1α as compared with CKD + DMEM BMCs in eight out of 10 rats (*P* = 0.014; Figure 1). Fewer apoptotic cells were found in CKD + pravastatin BMCs versus CKD + DMEM BMCs in eight out of 10 rats (*P* = 0.16; Figure 2). Pravastatin treatment of CKD BMCs did not alter mRNA expression of tumor necrosis factor alpha, endothelial NO synthase*,* protein kinase B*,* monocyte chemotactic protein 1 or vascular endothelial growth factor *genes* (Table 3). Of the 27 cytokines tested on a cytokine array, we could only detect expression of four cytokines. Pravastatin treatment of CKD BMCs decreased the secretion of proinflammatory CXCL5 and increased CXCL7, whereas secretion of L-selectin and soluble intracellular adhesion molecule was not different between DMEM-treated and pravastatin-treated CKD BMCs (Figure 3). Decreased CXCL5 secretion by CKD + pravastatin BMCs compared with DMEM-treated CKD BMCs was confirmed by enzyme-linked immunosorbent assay (60 ± 12 vs. 98 ± 31 pg/ml; *P* = 0.0045).

**Table 2:** **Bone marrow cell characteristics measured by fluorescence-activated cell sorting analysis**

*In vitro* pravastatin treated cells		
Bone marrow samples	DMEM (*n* = 10)	Pravastatin (*n* = 10)
Granulocytes	31.5 ± 2.6	30.8 ± 2.2
Lymphocytes	37.4 ± 2.6	38.6 ± 2.5
Monocytes	10.3 ± 1.9	10.9 ± 1.9
Stromal cells	7.9 ± 3.6	7.5 ± 3.5
Viability	96.6 ± 3.4	97.0 ± 2.5
Systemic *in vivo* pravastatin treatment recipients		
Bone marrow samples	CKD (*n* = 6)	CKD + pravastatin (*n* = 5)
Granulocytes	23.6 ± 1.5	23.8 ± 1.3
Lymphocytes	31.76 ± 2.8	31.8 ± 2.5
Monocytes	21.3 ± 0.77	16.9 ± 1.3*
Stromal cells	6.04 ± 0.4	7.76 ± 0.50*
Viability	96.8 ± 0.4	97.7 ± 0.01*

Data presented as mean ± standard deviation. CKD, chronic kidney disease; DMEM, Dulbecco’s modified Eagle medium. *P <0.05 versus CKD.

**Table 3:** **Gene expression in chronic kidney disease bone marrow cells not changed after** ***in vitro*** **treatment with pravastatin**

	**DMEM (** ***n*** **= 10)**	**Pravastatin (** ***n*** **= 10)**	***P*** **value**
TNFα	1.000 ± 1.290	0.868 ± 0.969	0.7504
eNOS	1.000 ± 4.609	2.161 ± 2.874	0.5422
PKB	1.000 ± 0.889	0.905 ± 0.5512	0.6906
MCP-1	1.000 ± 1.254	1.191 ± 0.6933	0.6101
VEGF	1.000 ± 0.566	0.687 ± 0.464	0.0807

Data presented as mean ± standard deviation, expressed as fold change. DMEM, Dulbecco’s modified Eagle medium; eNOS, endothelial nitric oxide synthase; MCP-1, monocyte chemotactic protein 1; PKB, protein kinase B; TNFα, tumor necrosis factor alpha; VEGF, vascular endothelial growth factor.

### *In vivo* experiments

#### *Ex vivo* pravastatin pre-treatment of CKD BMC improves *in vivo* therapeutic efficacy

As compared with healthy + DMEM BMC recipients, CKD + DMEM BMC recipients had a 38% lower GFR and 45% lower ERPF at week 12. However, in CKD + pravastatin BMC recipients the GFR and ERPF were not significantly different compared with healthy + DMEM BMC recipients (Table 4). Terminal mean arterial pressure was 20 mmHg lower in CKD + pravastatin BMC recipients compared with CKD + DMEM BMC recipients and not different from either healthy + DMEM BMC recipients or healthy + pravastatin BMC recipients (Table 4). Hematocrit was lower in CKD + DMEM BMC recipients versus healthy + DMEM BMC recipients, but higher in CKD + pravastatin BMC recipients versus CKD BMC recipients (Table 4). The filtration fraction was higher in CKD BMC recipients versus healthy BMC recipients, whereas in CKD + pravastatin BMC recipients the filtration fraction was not significantly different versus healthy + DMEM BMC recipients (Table 4). Terminal body, kidney and heart weights did not differ (Table 4).

**Table 4:** **Terminal measurements of bone marrow cell recipients with** ***post hoc P*** **values**

**Week 12**	**Healthy + DMEM BMC recipients (** ***n*** **= 5)**	**Healthy + pravastatin BMC recipients (** ***n*** **= 5)**	**CKD + DMEM BMC recipients (** ***n*** **= 10)**	**CKD + pravastatin BMC recipients (** ***n*** **= 9)**	**CKD + DMEM BMC vs. healthy + DMEM BMC**	**CKD + DMEM BMC vs. CKD + pravastatin BMC**
Body weight (g)	376 ± 23	379 ± 26	359 ± 38	373 ± 22	ns	ns
Heart weight (g/100 g BW)	0.44 ± 0.07	0.44 ± 0.03	0.47 ± 0.08	0.44 ± 0.05	ns	ns
Kidney weight (g/100 g BW)	0.59 ± 0.07	0.56 ± 0.05	0.53 ± 0.06	0.55 ± 0.04	0.063	ns
MAP (mmHg)	149 ± 28	146 ± 35	173 ± 21	153 ± 21	0.095	0.091
GFR (μl/minute/100 g)	371 ± 68	352 ± 119	237 ± 159	261 ± 149	0.057	ns
ERPF (μl/minute/100 g)	1538 ± 224	1388 ± 502	844 ± 626*	1157 ± 359	<0.05	ns
Hematocrit	0.45 ± 0.01	0.44 ± 0.01	0.42 ± 0.03*	0.46 ± 0.02^†^	<0.05	<0.05
FF (%)	24.1 ± 2.4	26.2 ± 4.7	29.9 ± 4.5*	26.4 ± 3.7	<0.05	0.071

Data presented as mean ± standard deviation. BMC, bone marrow cell; BW, body weight; CKD, chronic kidney disease; DMEM, Dulbecco’s modified Eagle medium; ERPF, effective renal plasma flow; FF, filtration fraction; GFR, glomerular filtration rate; MAP, mean arterial pressure. *P <0.05 compared with healthy BMC recipients, ^†^ *P <*0.05 compared with CKD BMC recipients. Trends (*P* <0.1) are only indicated with the exact *P* value.

**Figure 4:**
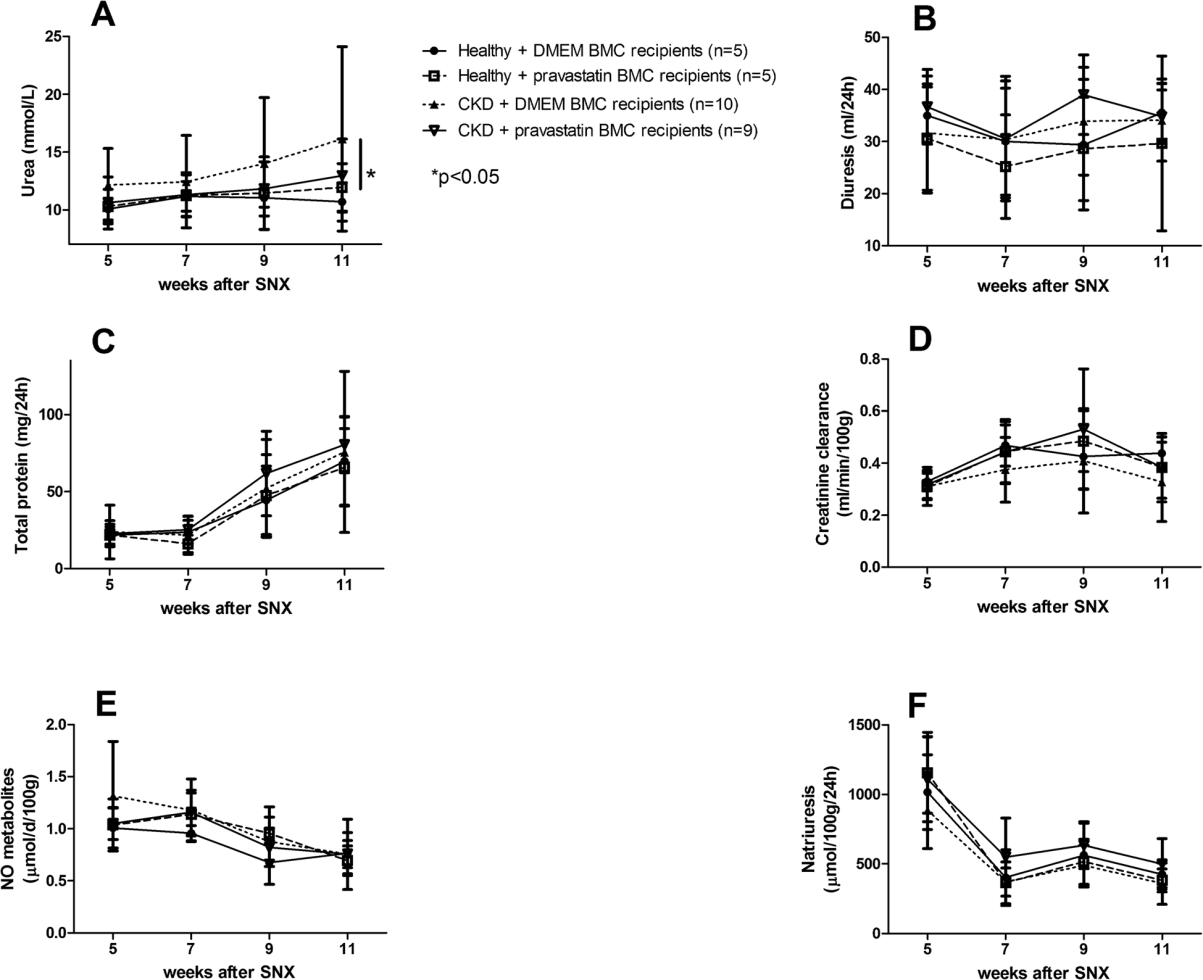
Effects of *ex vivo* exposure of healthy and chronic kidney disease bone marrow cell**s** to pravastatin or DMEM on longitudinal plasma and urinary variables of chronic kidney disease recipients. **(A)** Urea. **(B)** Diuresis. **(C)** Proteinuria. (**D)** Creatinine clearance. **(E)** Nitric oxide (NO) metabolites. **(F)** Natriuresis. Week 5 represents 1 week before BMC injection. Data presented as mean ± standard deviation. BMC, bone marrow cell; BW, body weight; CKD, chronic kidney disease; DMEM, Dulbecco’s modified Eagle medium; SNX, subtotal nephrectomy.

At week 11, plasma urea was higher in CKD + DMEM BMC recipients compared with healthy + DMEM BMC recipients (16.1 ± 7.9 vs. 10.7 ± 1.7 mmol/l, *P* < 0.05), whereas CKD + pravastatin BMC recipients (12.9 ± 3.2 mmol/l) were not significantly different from healthy + DMEM BMC recipients (10.7 ± 1.7 mmol/l) (Figure 4A). Diuresis, proteinuria, creatinine clearance, and excretion of NO metabolites were not significantly different between groups, but from weeks 7 to 11 natriuresis was higher in CKD + pravastatin BMC recipients versus CKD + DMEM BMC recipients (1,680 ± 473 vs. 1,176 ± 233; *P* <0.05; Figure 4B,C,D,E,F).

**Figure 5:**
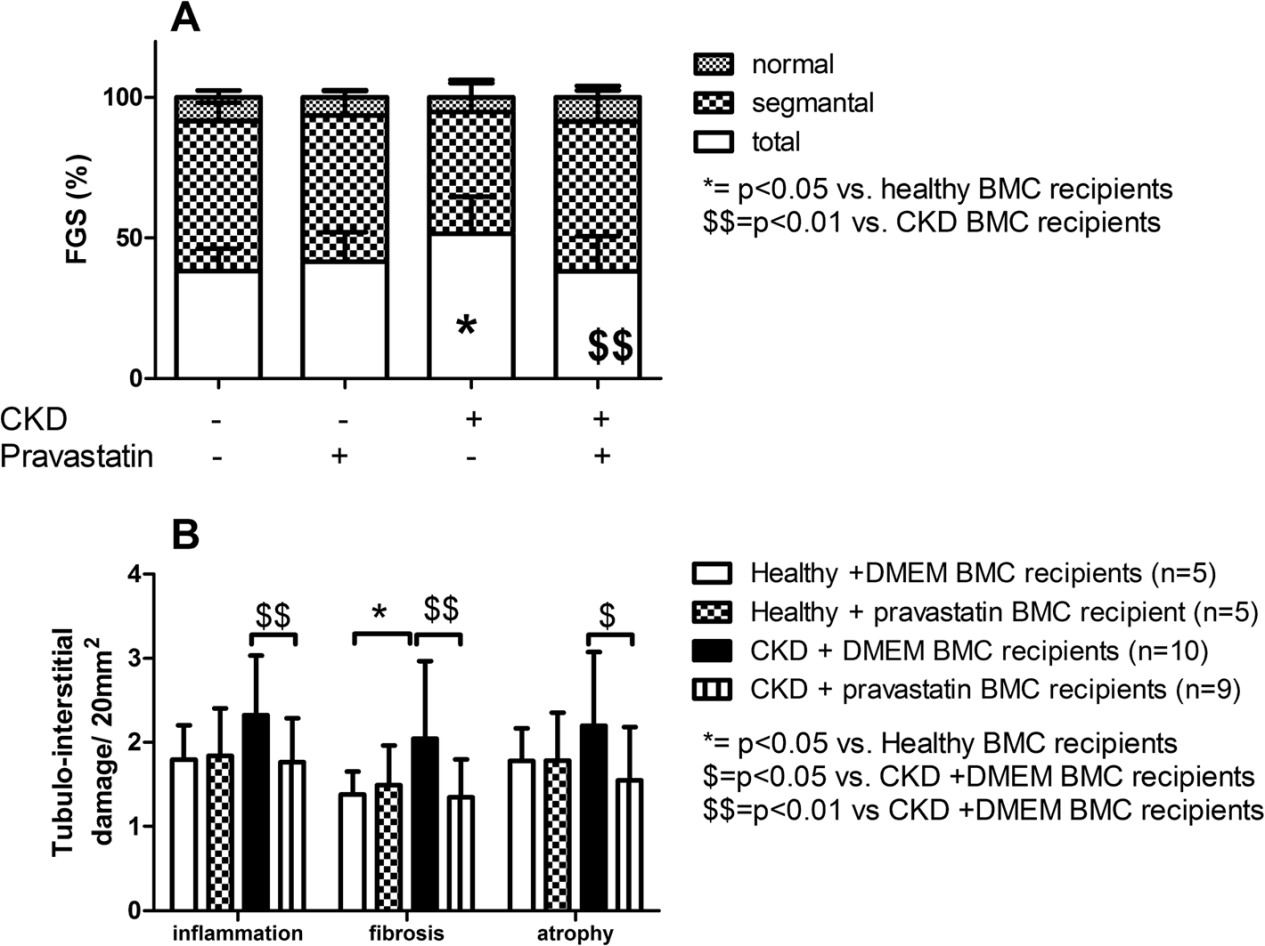
Effects of *ex vivo* exposure of healthy and chronic kidney disease bone marrow cell**s** to pravastatin on renal morphology in chronic kidney disease recipients. **(A)** Focal glomerulosclerosis. **(B)** Tubulo-interstitial damage. CKD rats received healthy + DMEM BMCs (*n* = 5), healthy + pravastatin BMCs (*n* = 5), CKD + DMEM BMCs (*n* = 10) or CKD + pravastatin BMCs (*n* = 9). *P <0.1 versus healthy BMC recipients, $*P* <0.1 and $$*P* <0.05 versus CKD + DMEM BMC recipients. BMC, bone marrow cell; CKD, chronic kidney disease; DMEM, Dulbecco’s modified Eagle medium; FGS, focal glomerulosclerosis.

In all CKD groups, only 10% of glomeruli were completely normal, confirming severe kidney injury. The number of totally sclerotic glomeruli was higher in CKD + DMEM BMC recipients versus healthy + DMEM BMC recipients (*P* <0.05). Comparing the number of partly and totally sclerotic glomeruli between CKD + DMEM BMC recipients and CKD + pravastatin BMC recipients revealed a favorable shift to better preserved glomeruli in CKD + pravastatin BMC recipients (*P* <0.05; Figure 5A). Tubular interstitial inflammation, tubular atrophy and interstitial fibrosis were all lower in CKD + pravastatin BMC recipients versus CKD + DMEM BMC recipients (Figure 5B). The number of glomerular inflammatory cells (ED1^+^ and CD3^+^) tended to be higher in CKD + DMEM BMC recipients compared with all other groups. Tubular CD3^+^ influx was most abundant in CKD + DMEM BMC recipients (Table 5). Cardiac fibrosis tended to be more abundant in CKD + DMEM BMC recipients compared with healthy BMC + DMEM recipients, as reported previously [23]. In CKD + pravastatin BMC recipients, cardiac fibrosis tended to be reduced compared with CKD + DMEM BMC recipients (15.3 ± 7.6 vs. 20.5 ± 4.7%; *P* = 0.085). Healthy + pravastatin BMCs did not influence cardiac fibrosis (healthy + DMEM BMC recipients: 16.7 ± 6.5 vs. healthy + pravastatin BMC recipients: 16.8 ± 6.7%).

**Table 5:** **Glomerular and tubular histological characteristics after** ***ex vivo*** **pravastatin pre-treatment**

	**Healthy + DMEM BMC recipients (** ***n*** **= 5)**	**Healthy + pravastatin BMC recipients (** ***n*** **= 5)**	**CKD + DMEM BMC recipients (** ***n*** **= 10)**	**CKD + pravastatin BMC recipients (** ***n*** **= 9)**
**Glomerular**				
CD3	0.68 ± 0.34	0.72 ± 0.33	0.76 ± 0.19	0.67 ± 0.27
ED-1	7.4 ± 1.0	5.0 ± 2.9	10.5 ± 5.5	7.9 ± 1.9
Ki67	8.0 ± 0.76	6.4 ± 1.7	7.8 ± 1.8	7.8 ± 2.3
TUNEL	2.6 ± 1.3	2.1 ± 1.3	7.0 ± 4.6	4.4 ± 3.7
γH2AX	1.5 ± 1.6	0.6 ± 0.4	1.2 ± 0.8	1.4 ± 0.8
GFP^+^	7.0 ± 6.6	2.5 ± 0.7	2.3 ± 1.9	5.2 ± 2.9
JG12	42.5 ± 10	44.4 ± 9.9	42.8 ± 12.5	48.1 ± 9.0
**Tubular**				
CD3	81 ± 11	96 ± 45	122 ± 59	98 ± 27
TUNEL	35 ± 24	65 ± 59	147 ± 116	95 ± 79
γH2AX	5.0 ± 1.6	4.3 ± 2.0	6.1 ± 2.8	5.3 ± 1.5
GFP^+^	83 ± 82	31 ± 24	23 ± 20	50 ± 38

Data presented as mean ± standard deviation per 50 glomeruli or 20 tubular fields. BMC, bone marrow cell; CKD, chronic kidney disease; DMEM, Dulbecco’s modified Eagle medium; GFP, green fluorescent protein; TUNEL, terminal deoxynucleotidyl transferase dUTP nick end labeling.

The number of proliferating glomerular cells (Ki67^+^), endothelial cells (JG12^+^), and apoptotic cells (TUNEL^+^) were not significantly different between groups (Table 5). No difference was found in the number of tubular apoptotic (TUNEL^+^) cells, cells expressing DNA damage repair marker γH2AX^+^ (Table 5), or the number of eGFP^+^ cells. As shown previously, eGFP^+^ cells were detected in the remnant kidney and heart but only in low numbers. No integration of these cells was observed and most were attached to the endothelial lining of small vessels [1]. No differences were observed in blood counts (Table 6).

**Table 6:** **Blood cell counts**

***Ex vivo*** **pravastatin pretreatment experiment recipients**				
**Blood samples**	**Healthy + DMEM BMC recipients (** ***n*** **= 5)**	**Healthy + pravastatin BMC recipients (** ***n*** **= 5)**	**CKD + DMEM BMC recipients (** ***n*** **= 10)**	**CKD + pravastatin BMC recipients (** ***n*** **= 9)**
White blood cells	5.86 ± 1.20	5.52 ± 0.93	4.63 ± 1.05	5.42 ± 1.18
Lymphocytes	3.30 ± 1.35	2.54 ± 0.29	2.60 ± 0.86	2.94 ± 0.51
Midpopulation	1.58 ± 0.29	1.56 ± 0.31	1.18 ± 0.28	1.59 ± 0.45
Granulocytes	0.98 ± 0.45	1.38 ± 0.53	0.84 ± 0.35	1.08 ± 0.52
Red blood cells	6.66 ± 0.40	6.59 ± 0.36	6.24 ± 0.59	6.63 ± 0.23
Hemoglobin	8.06 ± 0.47	7.90 ± 0.42	7.63 ± 0.72	7.86 ± 0.29
Hematocrit	0.33 ± 0.05	0.33 ± 0.019	0.31 ± 0.029	0.33 ± 0.010
Mcv	49.08 ± 0.83	50.08 ± 1.08	50.10 ± 2.09	49.35 ± 0.78
Mchc	24.38 ± 0.22	23.94 ± 0.38	24.18 ± 0.43	24.08 ± 0.36
Rdw	15.44 ± 0.43	15.22 ± 0.29	15.63 ± 1.125	15.151 ± 0.818
Platelets	736 ± 68	697 ± 49	634 ± 99	699 ± 55
**Systemic** ***in vivo*** **pravastatin treatment**				
**Blood samples**	**CKD (n = 6)**	**CKD + pravastatin (** ***n*** **= 5)**		
White blood cells	4.81 ± 0.89	4.50 ± 1.16		
Lymphocytes	2.87 ± 0.59	2.90 ± 0.97		
Midpopulation	1.27 ± 0.39	0.90 ± 0.19		
Granulocytes	0.65 ± 0.16	0.68 ± 0.13		
Red blood cells	6.38 ± 0.22	6.25 ± 0.15		
Hemoglobin	7.58 ± 0.32	7.52 ± 0.18		
Hematocrit	0.32 ± 0.01	0.31 ± 0.01		
Mcv	49.37 ± 0.79	49.96 ± 0.32		
Mchc	24.05 ± 0.25	24.14 ± 0.23		
Rdw	15.13 ± 0.63	15.14 ± 0.68		
Platelets	681 ± 42	666 ± 41		

Data presented as mean ± standard deviation. BMC, bone marrow cell; CKD, chronic kidney disease; DMEM, Dulbecco’s modified Eagle medium. Mcv, Mean Corpuscular Volume; Mchc, Mean Corpuscular Hemoglobin Concentration; Rdw, Relative Distribution Width.

### Systemic *in vivo* treatment of CKD rats with pravastatin does not reduce CKD progression

**Figure 6:**
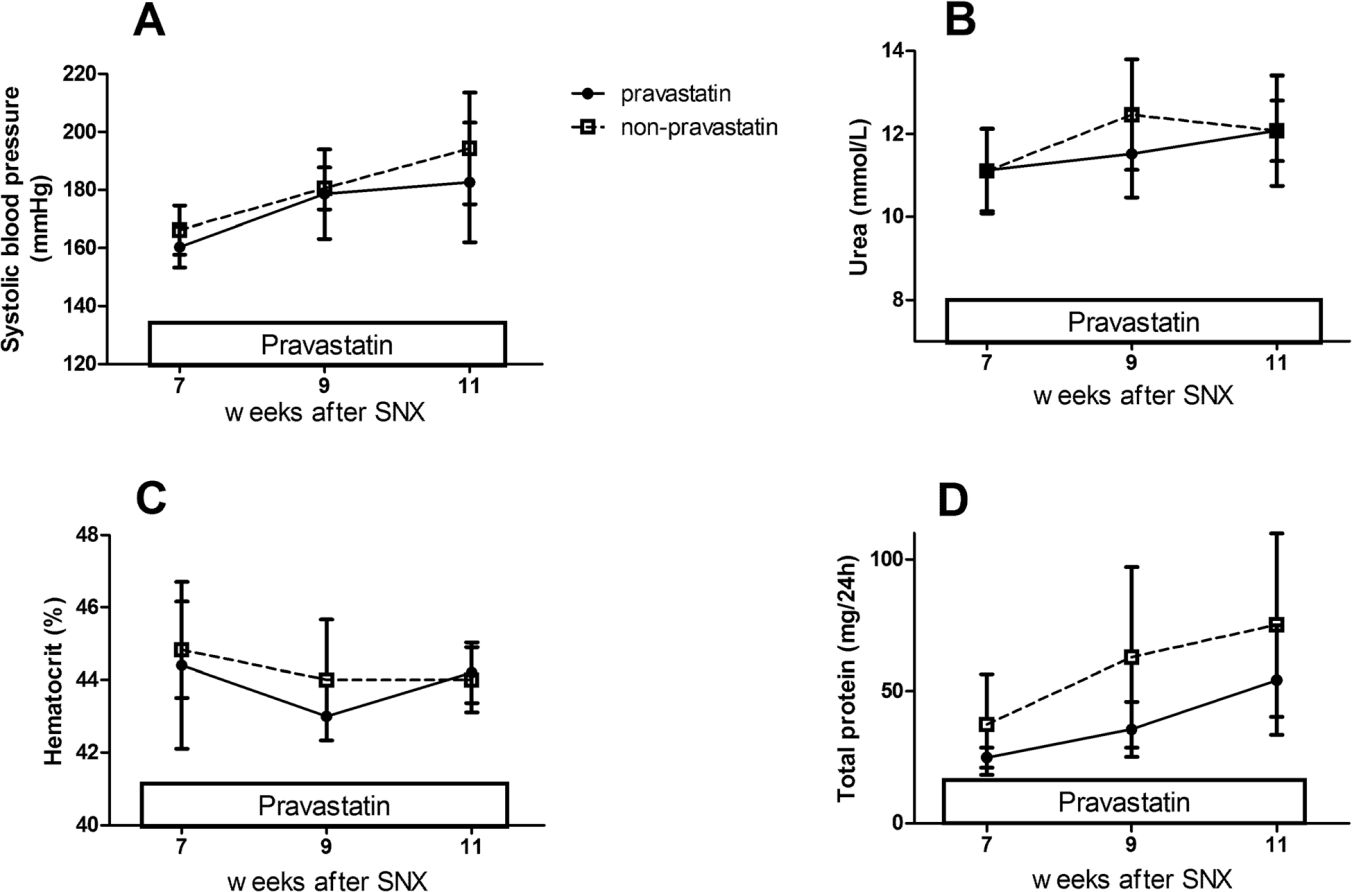
Effects of systemic *in vivo* exposure to pravastatin on longitudinal variables in chronic kidney disease recipients. **(A)** Systolic blood pressure. **(B)** Urea. **(C)** Hematocrit. **(D)** Proteinuria. SNX, subtotal nephrectomy.

CKD rats developed hypertension, mild uremia, anemia and proteinuria (Figure 6). Systemic 6-week *in vivo* pravastatin treatment did not influence the body weight, GFR, ERPF, filtration fraction or hematocrit (Table 7). Furthermore, no differences in blood counts (Table 6), focal glomerulosclerosis and tubulo-interstitial damage (Table 8), cardiac fibrosis (3.54 ± 2.20 vs. 4.79 ± 2.71%) or NO metabolite excretion (Figure 6) were observed between CKD and CKD + pravastatin rats. Cholesterol did not differ between CKD + pravastatin-treated rats and CKD rats; however, triglycerides were significantly lower in CKD + pravastatin-treated rats (Table 7). Systemic pravastatin decreased the number of monocytes and increased the stromal cell population in the BM (Table 2) and increased hepatic HMGCR mRNA expression (Table 7).

**Table 7:** **Terminal measurements after systemic** ***in vivo*** **pravastatin treatment**

**Week 12**	**CKD (** ***n*** **= 6)**	**CKD + pravastatin (** ***n*** **= 5)**
Body weight (g)	357 ± 24	369 ± 16
Heart weight (g/100 g BW)	0.45 ± 0.02	0.42 ± 0.03*
Kidney weight (g/100 g BW)	0.57 ± 0.02	0.57 ± 0.04
MAP (mmHg)	168 ± 19	164 ± 19
GFR (μl/minute/100 g)	340 ± 60	332 ± 41
ERPF(μl/minute/100 g)	1335 ± 291	1221 ± 189
Hematocrit	0.41 ± 0.02	0.40 ± 0.02
FF (%)	28 ± 3	26 ± 2
Cholesterol (mmol/l)	2.89 ± 0.53	2.73 ± 0.46
Triglycerides (mmol/l)	1.42 ± 0.38	0.70 ± 0.37*
Fold change hepatic HMGCR mRNA expression	1.000 ± 0.56	1.584 ± 0.65

Data presented as mean ± standard deviation. BW, body weight; CKD, chronic kidney disease; ERPF, effective renal plasma flow; FF, filtration fraction; GFR, glomerular filtration rate; HMGCR, 3-hydroxy-3-methyl-glutaryl-CoA reductase; MAP, mean arterial pressure. *P <0.05 compared with CKD.

**Table 8:** **Longitudinal, terminal and histological measurements after** ***in vivo*** **pravastatin treatment**

	**CKD (** ***n*** **= 6)**	**CKD + pravastatin (** ***n*** **= 5)**
Focal glomerulosclerosis (%)		
Normal	32.9 ± 7.6	23.4 ± 13.1
Partial	48.1 ± 10.6	62.7 ± 10.4
Total	19.0 ± 6.7	14.0 ± 15.8
Tubulo-interstitial damage		
Fibrosis	0.78 ± 0.31	0.85 ± 0.14
Inflammation	1.72 ± 0.54	1.66 ± 0.22
Atrophy	1.09 ± 0.65	0.56 ± 0.09

Data presented as mean ± standard deviation. CKD, chronic kidney disease.

## Discussion

The present study demonstrates for the first time that BMC dysfunction in CKD can be reversed by short-term (2 hours) pretreatment with pravastatin outside the CKD environment and that this effect persists when the cells are returned to the CKD environment, providing augmented therapeutic efficacy *in vivo.*

Our recent studies have shown that a single injection of healthy BMCs in rats with established CKD slowed progression of the disease, probably via paracrine actions. Less disease progression was characterized by increased glomerular capillary density and less sclerosis. Injection of BMCs derived from CKD rats was less effective [1], suggesting that CKD induces alterations in (paracrine) functions of BMC which reduce endothelial regenerative capacity and efficacy of CKD BMC therapy in rats. Previously, statins have been reported to exert beneficial effects on endothelial as well as on BM-derived EPC and MSC function both after *in vitro* incubation and after systemic *in vivo* treatment [5,7-9]. Here we show that short-term *ex vivo* pretreatment with pravastatin reverses paracrine dysfunction in BMCs obtained from rats with established CKD, resulting in preserved renal morphology in recipient rats with CKD.

The role of transdifferentiation and incorporation of BMCs in enhancing tissue regeneration has been questioned. BMCs appear to have a supportive function, secreting growth factors and cytokines, thereby stimulating resident cells to engage in regeneration [24]. Using a cytokine array we showed that short-term pravastatin pretreatment influences paracrine factor secretion by BMCs. CKD + pravastatin BMCs significantly decreased expression of the proinflammatory chemokine CXCL5, which was shown to be involved in the recruitment and activation of polymorphonuclear neutrophils and in stimulation of local production of cytokines that have proapoptotic effects [25]. Our findings are in accordance with previous reports that atorvastatin dose-dependently inhibits basal CXCL5 production in human umbilical vein endothelial cells [26] and that simvastatin inhibited CXCL5 release from peripheral blood mononuclear cells [27]. In our experiments, renal influx of inflammatory cells was lowered in CKD + pravastatin BMC recipients versus CKD + DMEM BMC recipients. In glomeruli, there was a trend towards more ED-1^+^ macrophages and CD3^+^ T cells in CKD + DMEM BMC recipients and the influx in CKD + pravastatin BMC recipients was lower and comparable with both healthy + DMEM BMC recipients and healthy + pravastatin BMC recipients, suggesting an *in vivo* anti-inflammatory effect of *in vitro* pravastatin-pretreated BMCs. Increased paracrine function can explain why we did not observe an increase in the number of eGFP^+^ cells in the remnant kidney. Our observations that few eGFP^+^ BMCs were found in kidney sections of all recipients, and that those found were in close proximity to the microvasculature but did not differentiate into endothelial cells, are consistent with paracrine actions of BMCs reported by others [28,29].

Impaired BMC function in CKD rats is consistent with clinical studies reporting impaired function of BM-derived EPCs obtained from CKD patients [30,31]. We showed previously that culturing healthy BM mononuclear cells in uremic serum caused reduced outgrowth of EPCs, suggesting that uremic serum contains either impairing toxins or lacks essential stimulants to support EPC function [31]. Indeed, better *in vivo* removal of uremic toxins in CKD patients has been shown to improve EPC function [32,33]. However, culturing of CKD BMCs in nonuremic conditions *in vitro* could not reverse the impairment in outgrowth towards EPCs or EPC function [31,34]. In our study, 2 hours of incubation in DMEM outside a CKD environment did not reverse BMC dysfunction. Importantly, our experiments show that 2 hours of incubation with pravastatin improved rat CKD BMC function, which may have important clinical consequences if confirmed in human CKD. Induction of improvement in CKD BMC function by HMGCR inhibition within 2 hours is remarkable but seems consistent with previous reports showing that short-term statin incubation (<10 minutes) induces a rapid elevation of NO production in endothelial cells [35] and rapid (<30 minutes) induction of Akt-mediated phosphorylation of endothelial NO synthase leading to NO production [36].

Filtration fraction was increased in CKD + DMEM BMC recipients versus healthy BMC recipients (*P* <0.05), indicating a less well preserved glomerular structure. Mean arterial pressure was 20 mmHg lower in rats that received CKD + pravastatin CKD BMCs or healthy + DMEM BMCs compared with recipients that received CKD + DMEM BMCs, and healthy + DMEM BMC, healthy + pravastatin BMC and CKD + pravastatin BMC recipients tended to have a higher natriuresis per 24 hours versus CKD + DMEM BMC recipients. Blood pressure lowering of 20 mmHg has major renal and cardiovascular implications such as decreased risk of stroke [37], myocardial infarction, cardiac failure [38] and peripheral arterial disease and an increased life expectancy [39]. Consistently, we observed significantly less glomerulosclerosis, tubular inflammation, atrophy and fibrosis in remnant kidneys of rats that received CKD + pravastatin BMCs compared with CKD + DMEM BMC recipients. Cardiac fibrosis tended to be increased in CKD + DMEM BMC recipients compared with CKD + pravastatin BMC recipients. *Ex vivo* pravastatin treatment did not further improve renal function or structure or decrease cardiac fibrosis in healthy + pravastatin BMC recipients compared with healthy + DMEM BMC recipients, whereas in CKD + pravastatin BMC recipients cardiac fibrosis was significantly decreased compared with CKD + DMEM BMC recipients, indicating that pravastatin specifically corrected CKD BMC function. We have not compared different statins in our experimental setup, and therefore our study does not allow conclusions as to whether this phenomenon is a specific effect of pravastatin or a generic statin effect.

Interestingly, 6 weeks of systemic *in vivo* treatment with pravastatin did not influence CKD progression or renal fibrosis in our model of established CKD. Although cholesterol-lowering effects of statins do not occur in rodents, pleiotropic effects have been reported [40] such as reduced inflammation and oxidative stress, enhanced endothelial function and increased mobilization and function of EPCs. Some older studies showed beneficial effects of statins on renal function and morphology in experimental CKD, although others reported harmful effects such as induction of renal fibrosis [41,42]. Recently, Geng and colleagues performed a meta-analysis on the effect of statins on renal function (estimated GFR and proteinuria) in patients with CKD. The analysis showed that the beneficial effect of statins on renal function may be dose and time dependent and that statins are well tolerated in patients with National Kidney Foundation Kidney Disease Outcomes Quality Initiative stages 1 to 3. However, the effect of statins on renal function in National Kidney Foundation Kidney Disease Outcomes Quality Initiative stages 4 and 5 remains controversial [43]. The lack of effect of statin treatment in our *in vivo* study cannot be explained by insufficient dosing of pravastatin. Similar statin doses have previously been shown to increase EPC mobilization in mice [44]. Furthermore, we observed a significant decrease in triglycerides and an increase in HMGCR mRNA expression after systemic pravastatin treatment, which indicates that the dose was sufficient to affect the mevalonate pathway, and is in agreement with Zager and colleagues [45].

## Conclusions

Short-term pretreatment of CKD BMCs with pravastatin reversed CKD BMC dysfunction and improved the cells’ therapeutic efficacy *in vivo*. Our data suggest that this is due to an improvement in their paracrine profile*.* In contrast, systemic *in vivo* pravastatin treatment did not attenuate the progressive course of CKD. Our findings have relevance for potential clinical application of BMC therapy in patients with CKD as clinical application would involve autologous – and thus CKD – BMCs to avoid immunological reactions. If confirmed for human CKD BMCs, our findings will provide a basis for development of clinical trials and application of autologous BMC-based therapies in human CKD.

## Abbreviations

BM: bone marrow
BMC: bone marrow cell
CKD: chronic kidney disease
CXCL: chemokine (C-X-C motif) ligand
DMEM: Dulbecco’s modified Eagle medium
eGFP: enhanced green fluorescent protein
EPC: endothelial progenitor cell
ERPF: effective renal plasma flow
GFR: glomerular filtration rate
HMGCR: 3-hydroxy-3-methyl-glutaryl Coenzyme A reductase
MSC: mesenchymal stem cell
NO: nitric oxide
SDF1α: stromal derived factor 1 alpha
TUNEL: terminal deoxynucleotidyl transferase dUTP nick end labeling
